# Effect of polyphenols from *Syringa vulgaris* on blood stasis syndrome

**DOI:** 10.3164/jcbn.20-55

**Published:** 2020-06-09

**Authors:** Hisae Oku, Maki Maeda, Fumika Kitagawa, Kyoko Ishiguro

**Affiliations:** 1 School of Pharmacy and Pharmaceutical Sciences, Mukogawa Women’s University, Koshien Kyuban-cho 11-68, Nishinomiya, Hyogo 663-8179, Japan

**Keywords:** *Syringa vulgaris*, blood flow, stagnant blood syndrome, hen egg white lysozyme, antioxidant

## Abstract

In this study, we employed a previously developed *in vivo* assay system to determine whether the flowers and leaves of *Syringa vulgaris* (*S. vulgaris*; commonly known as “lilac”) can prevent blood stasis syndrome, known as *oketsu* in Japanese. This syndrome is considered an important pathology in traditional Chinese and Japanese medicine, and is related to diseases such as peripheral vascular disorders, blood vessel inflammation, and platelet aggregation, whose severities are augmented owing to lipid peroxidation, free radicals, and oxidative stress. The assay system employed in this study monitored the blood flow decrease in the tail vein of mice subjected to sensitization with hen egg white lysozyme. Through bioassay-guided fractionation of different *S. vulgaris* extracts, five polyphenols were isolated and identified. Among them, quercetine 3-glucoside, quercetin 3-rutinoside, and acteoside were identified as active compounds, as they significantly mitigated blood flow reduction. These findings indicate that the polyphenols obtained from *S. vulgaris* could be useful for preventing *oketsu* and improve the quality of life of individuals with disorders and diseases such as gynecopathy, cold sensitivity, poor circulation, allergy, and lifestyle-related diseases.

## Introduction

The leaves of *Syringa vulgaris* (*S. vulgaris*; Oleaceae family), commonly known as “lilac”, are used in traditional Chinese medicine for its analgesic, pyretolysis, anti-inflammatory, and stomachic properties; scientific data confirmed the antioxidant, anti-inflammatory, and antipyretic properties of *S. vulgaris*.^(1–4)^ Several chemical constituents including flavonoids, iridoids, and cinnamic acid derivatives have been identified in the flowers and leaves of *S. vulgaris*.^(1,5,6)^ Part of these compounds have been reported to show inhibitory effects on lipid peroxidation, free radical formation, and oxidative stress.^(7–9)^ These three phenomena are important factors affecting blood stasis syndrome, because they lead to severe peripheral vascular disorders, blood vessels inflammation, and platelet aggregation. Blood stasis syndrome, known as *oketsu* in Japanese, is an important underlying pathology related to blood circulation disorders and inflammation in the traditional Chinese and Japanese medicine. It is one of the leading causes of gynecopathy, cold sensitivity, poor circulation, and shoulder discomfort. Currently, this syndrome has been linked with atopic dermatitis, cerebrovascular and cardiovascular diseases, and lifestyle-related diseases such as diabetes and high blood pressure. The development of novel drugs to treat *oketsu* can improve the quality of life of individuals with these symptoms. In a previous study, we established a peripheral blood circulatory disease model in mice that simulates *oketsu*. Using this model, we developed an *in vivo* assay system to identify novel substances from natural sources that could prevent blood stasis syndrome.^(10)^ In this system, the blood flow in the tail vein microcirculation of mice subjected to sensitization with hen egg white lysozyme (HEL) is monitored; a decrease in blood flow is considered to be caused by the contraction of peripheral blood vessels and an increase in the viscosity of blood, which resylts in changes in blood pressure and shortening of blood clotting time. This assay method was originally developed to investigate novel allergy-preventive substances by monitoring the blood flow decrease in allergy induction phase owing to HEL sensitization without the need for performing HEL allergy tests.^(11)^ By using the inhibitors of chemical mediators and factors that may be related to stagnant blood, we found that the decrease in blood flow is complexly regulated by various factors, including nitric oxide (NO), thromboxane (TX) A_2_, prostaglandin I_2_, granulocytic elastase (GE) and endothelin-1 (ET-1) together with cyclooxygenase (COX)-1 and -2, inducible nitric oxide synthase (iNOS), and constitutive nitric oxide synthase (cNOS).^(11,12)^ After further study of this phenomenon, we found a similarity between the blood flow decease in the model and the stagnant blood flow in *oketsu*.^(10)^ However, we could not prove that the decline in blood flow was related to the occurrence of an allergic reaction; we currently know that this phenomenon always occurs because of the presence of various antigens. Not only allergy-preventive drugs but also Kampo formulae (preparations based on a traditional Japanese medicine system that originated from ancient Chinese medicine) and crude drugs are being clinically used to treat *oketsu*. These drugs were found to address HEL-induced blood flow decrease but showed negligible effects when used for other purposes. By using the aforementioned assay method, we demonstrated that the active compounds of dried flowers of *Campsis grandiflora*, a traditional Chinese medicine used to treat stagnant blood and gynecopathies, such as menstrual and menopausal disorders, can be successfully employed to mitigate stagnant blood flow.^(13)^

In our continuing search for novel substances from natural sources, we have investigated the flowers and leaves of *S. vulgaris*. The medical applications of this plant for treating blood stasis syndrome have not been addressed in the literature yet. In the present study, we evaluated whether methanolic extracts from the flowers and leaves of *S. vulgaris* and the isolated compounds from these extracts can be used to reduce stagnant blood flow due to blood stasis syndrome.

## Materials and Methods

### General experimental procedure

The melting point was determined using a Yanagimoto micro melting point apparatus. The IR spectra were recorded on a Shimazu IR-435 spectrometer and UV absorption spectra on a Shimazu UV-160A spectrometer. ^1^H-NMR (500 MHz) and ^13^C-NMR (125 MHz) spectra were recorded on a JEOL JNM-ECP 500 spectrometer (TMS as the internal reference). MS was performed on a JMS-700 double-focusing spectrometer with xenon atom of kinetic energy equivalent to 6 kV at ion accelerating voltage.

### Plant materials

The flower and leaf of *S. vulgaris* were collected from the medicinal plant botanical garden in Mukogawa Women’s University (Nishinomiya, Japan).

### Extraction and isolation

Fresh flowers (6.4 g) and leaves (5.6 g) of *S. vulgaris* were extracted with methanol (MeOH) at room temperature, and then filtered. The obtained filtrates from the flowers (FSV) and leaves (LSV) were completely evaporated under vacuum, producing residues FSV (0.5 g) and LSV (0.7 g), respectively. Then, 104 g of LSV was suspended in water and extracted with ethyl acetate (AcOEt) and *n*-butanol (*n*-BuOH) to obtain AcOEt extract (25.8 g), an *n*-BuOH extract (26.8 g), and a H_2_O extract (49.7 g). The AcOEt extract was subjected to silica-gel column chromatography using a CHCl_3_-MeOH gradient elution to obtain ten fractions (Frs. A I–X). Fr. A VII (2.59 g) was repeatedly eluted by silica-gel column chromatography using CHCl_3_–MeOH gradient elution to obtain 10 fractions (Frs. B I–X). Fr. B VII (15.1 g), which was eluted with CHCl_3_–MeOH (10:1), was subjected to silica-gel column chromatography using AcOEt–MeOH gradient elution and then purified by gel filtration on Sephadex LH-20 using MeOH to yield compound **3** (4.1 mg). Fr. B VIII (32.9 g), which was eluted with CHCl_3_–MeOH (10:1–9:1), was purified by gel filtration on Sephadex LH-20 using MeOH to yield compound **1** (3.5 mg). The *n*-BuOH extract was subjected to silica-gel column chromatography using CHCl_3_–MeOH gradient elution to obtain seven fractions (Frs. C I–VII). Fr. C V (2.53 g), which was eluted with CHCl_3_–MeOH (4:1–2:1), was repeatedly eluted by silica-gel column chromatography using CHCl_3_–MeOH gradient elution to obtain compound **2** (7.0 mg). Fr. C VI (2.82 g), which was eluted with CHCl_3_–MeOH (2:1), was repeatedly eluted by silica-gel column chromatography using CHCl_3_–MeOH gradient elution to yield compound **4** (0.9 mg). Fr. C VI I (3.48 g), which was eluted with CHCl_3_–MeOH (2:1–1:1), was repeatedly eluted by silica-gel column chromatography using CHCl_3_–MeOH gradient elution to yield compound **5** (2.1 mg).

### Materials

HEL and complete Freund’s adjuvant (CFA) were purchased from Sigma-Aldrich, Co. LLC. (St. Louis, MO) and Difco Laboratories (Detroit, MI), respectively.

### Animals

Male ddY mice (SPF grade, 5-week-old) were obtained from Japan SLC, Inc. (Shizuoka, Japan) and housed at 24 ± 2°C. Food and water were provided *ad libitum*. All experiments were performed in accordance with the Guidelines for Animal Experiments of Mukogawa Women’s University.

### HEL sensitization

Immunization with HEL was performed as previously described, with slight modifications. Male ddY mice (5 weeks old) were sensitized subcutaneously with 50 µg of HEL in 50% CFA on day 0.^(10)^

### Blood flow measurement

Subcutaneous blood flow in the mouse tail was monitored using a contact type Laser Doppler Blood Flow Meter (FLO-C1; Neuroscience, Tokyo, Japan) as previously reported.^(10)^ Each mouse was prewarmed for 10 min at 36°C before the experiment and placed on a holder in a measuring chamber maintained at 36°C throughout the experiment. The blood flow was measured for 10 min without anesthetizing the mice. The results were expressed as mean ± SE of the percent of normal blood flow in each mouse, measured one day before the experiment.

### Assessment of blood flow improvement

Residues FSV and LSV (100 mg/kg) residues and the isolated compounds (10 mg/kg) were resuspended in water and administered orally to the mice on days 0 (1 h before sensitization), 3, and 6 of sensitization. Statistical significance was determined by comparison with the results from the HEL-sensitized mice (control group).

### Statistical analysis

All data are expressed as mean ± SE. All statistical analyses were conducted using the software GraphPad Prism 7. The results with a *p* value of <0.05 were considered statistically significant. A two-way analysis of variance (ANOVA) was used to test statistical differences in blood flow. When significant differences were identified, the data were further analyzed by Dunnett’s multiple range test coupled with a Bonferroni post hoc test for significant differences between each test group and the control group.

## Results

### Effect of oral administration of MeOH extracts of flowers and leaves of *S. vulgaris* on HEL-induced blood flow decrease

**Fig. 1:**
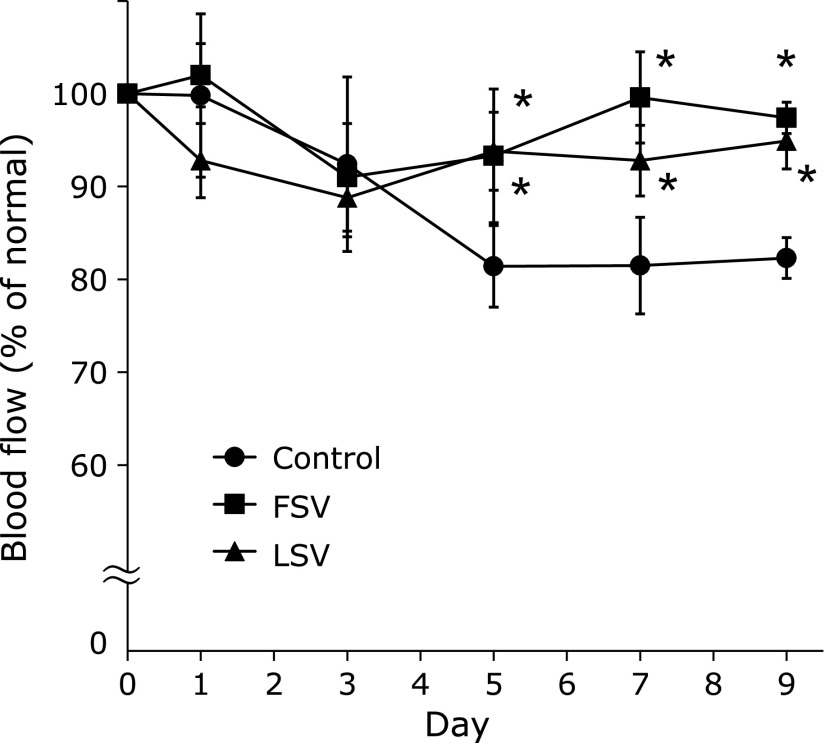
Effect of MeOH extract of *Syringa vulgaris* flowers (FSV) and leaves (LSV) on HEL-induced blood flow decrease. The extracts (100 mg/kg) were administered on days 0, 3, and 6 after sensitization. Each value represents the mean ± SE of five mice. *p<0.05 compared with the control group.

As shown in Fig. 1, the blood flow in HEL-sensitized mice of the control group gradually and significantly decreased, dropping to approximately 80% of the normal blood flow on day 9. The oral administration (100 mg/kg) of both FSV and LSV significantly (*p*<0.05) attenuated the decrease in blood flow. However, this treatment did not mitigate blood flow reduction in the mice without HEL-sensitization (data not shown). Thus, FSV and LSV inhibited the decrease in blood flow in the tail vein of mice subjected to HEL sensitization, indicating that the flowers and leaves of *S. vulgaris* can be used to prevent severe blood stasis syndrome. Using LSV, which was easy to obtain, we investigated the active compounds common to both FSV and LSV.

### The isolation and identification of polyphenols from LSV

**Fig. 2:**
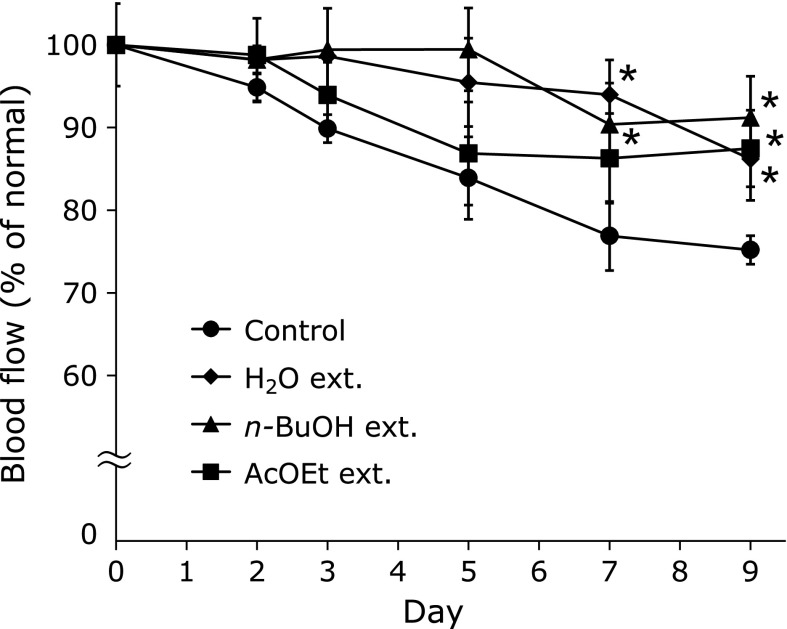
Effect of H_2_O, *n*-butanol (*n*-BuOH), and ethyl acetate (AcOEt) extracts obtained from LSV on HEL-induced blood flow decrease. The extracts (100 mg/kg) were administered on days 0, 3, and 6 after sensitization. Each value represents the mean ± SE of five mice. *p<0.05 compared with the control group.

**Fig. 3:**
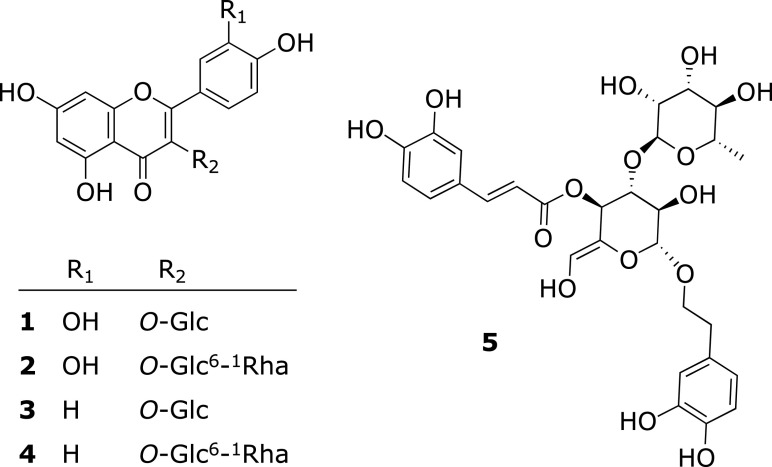
Chemical structures of compounds **1**–**5**.

Oral administration of the AcOEt, *n*-BuOH and H_2_O extracts (100 mg/kg) from LSV significantly attenuated the HEL-induced blood flow decrease after days 9, 7, and 7 of sensitization, respectively (Fig. 2). Through bioassay-directed fractionation, compounds **1** and **3** were isolated from the AcOEt extract and compounds **2**, **4**, and **5** were isolated from the *n*-BuOH extract. The isolated compounds were identified as quercetin 3-glucoside (**1**), quercetin 3-rutinoside (**2**), kaempferol 3-glucoside (**3**), kaempferol 3-rutinoside (**4**), and acteoside (**5**), by comparing their physical and spectroscopic data with standard samples (Fig. 3).

### Oral administration of isolated polyphenols from LSV

**Fig. 4:**
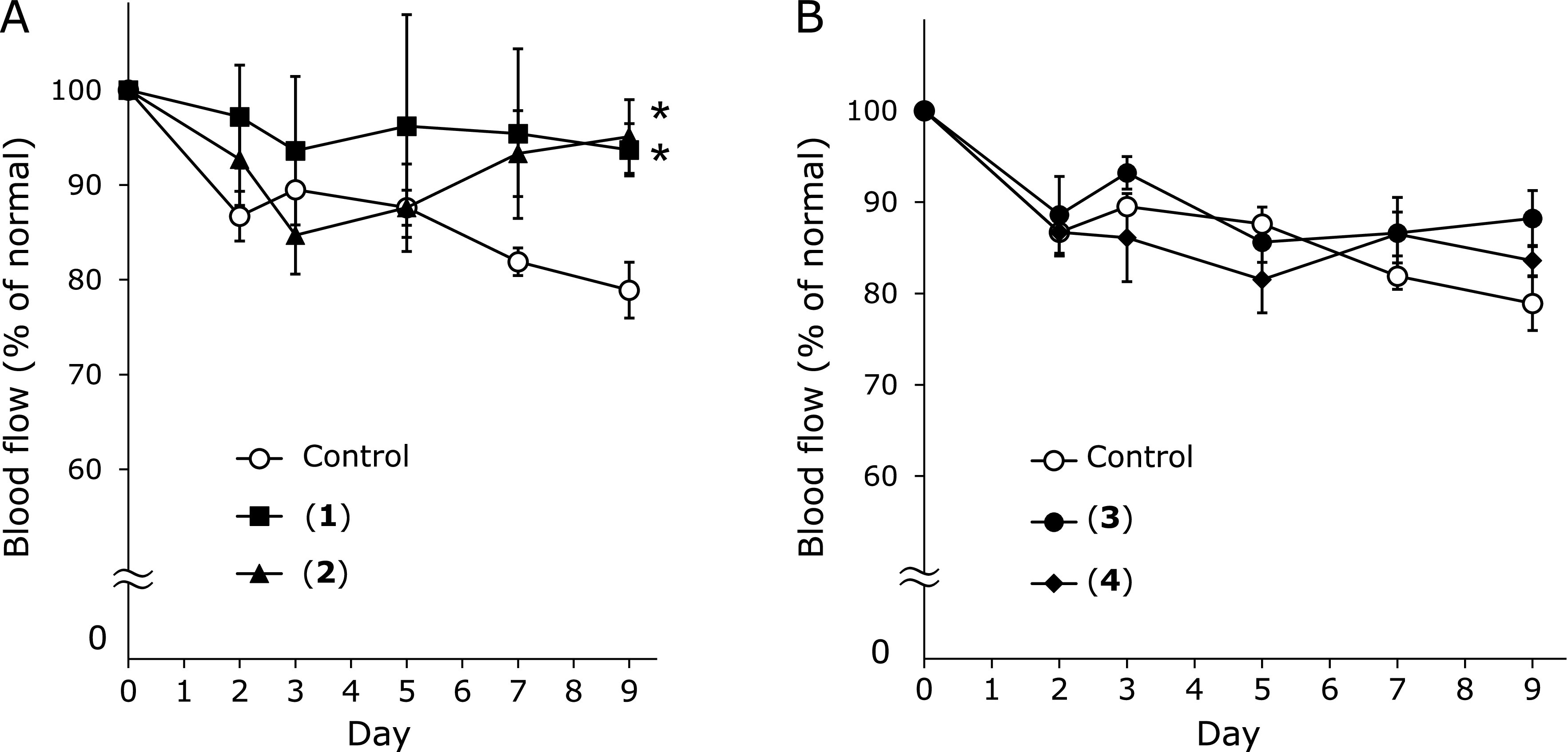
Effect of isolated compounds from LSV on HEL-induced blood flow decrease: (A) compounds quercetin 3-glucoside (**1**) and quercetin 3-rutinoside (**2**), and (B) kaempferol 3-glucoside (**3**) and kaempferol 3-rutinoside (**4**). The compounds (10 mg/kg) were administered on days 0, 3, and 6 after sensitization. Each value represents the mean ± SE of five mice. *p<0.05 compared with the control group.

Among the identified compounds, we previously demonstrated that **5** inhibited HEL-induced blood flow decrease.^(13)^ In the present study, the activity of compounds **1**–**4** was tested against HEL-induced blood flow decrease. As shown in Fig. 4, oral administration (10 mg/kg) of the compounds **1** and **2** significantly attenuated blood flow decrease compared with the results in the control group after day 9 of HEL-sensitization. However, compounds **3** and **4** did not attenuate blood flow. Compounds **1**–**4** did not affect blood flow in normal mice (data not shown). These findings indicate that compounds **1** and **2**, the active substances in LSV, can prevent blood flow decrease. Thus, compounds **1** and **2** were considered the main active compounds in the AcOEt and *n*-BuOH extracts, respectively.

## Discussion

**Fig. 5:**
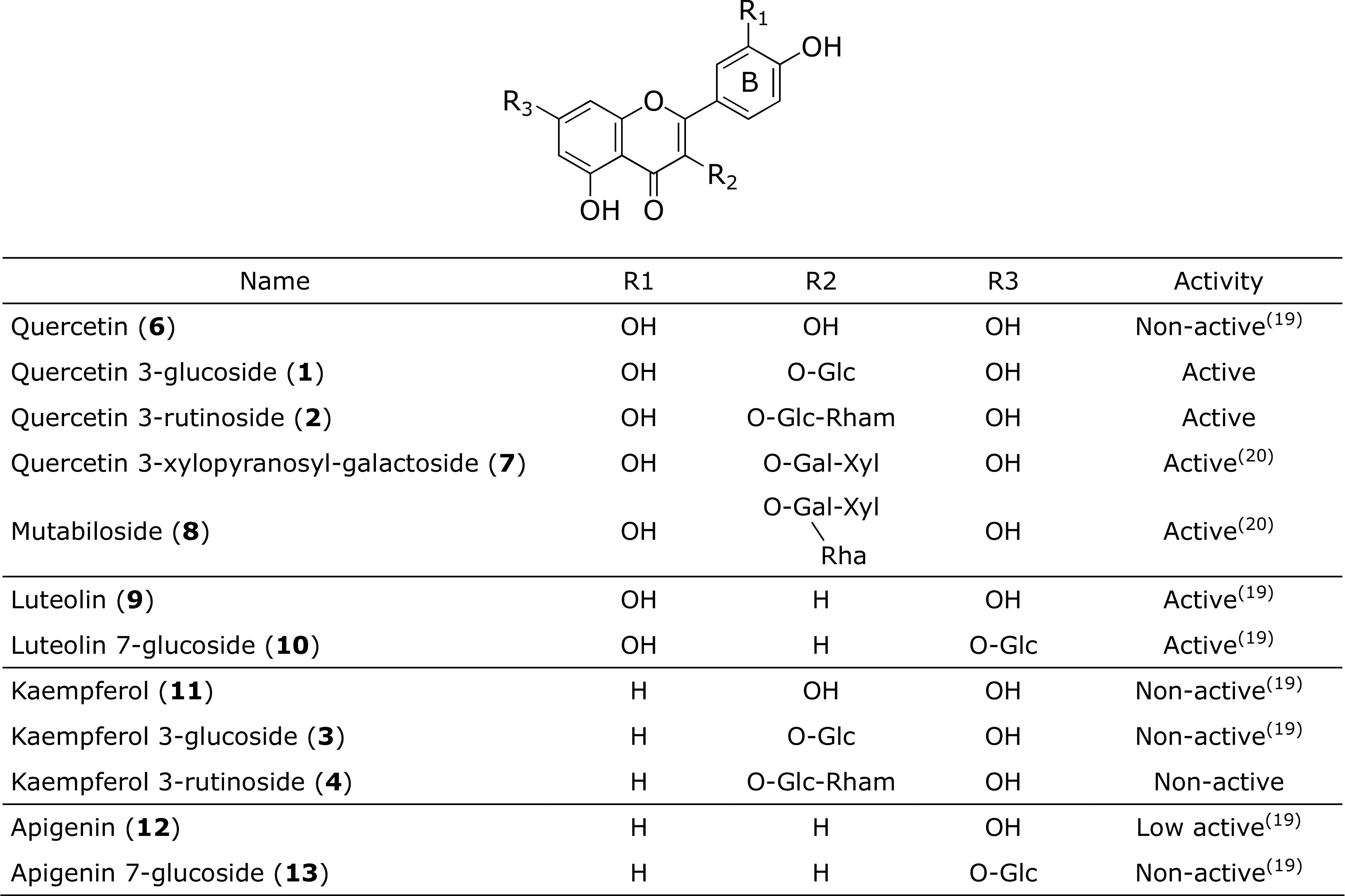
Effects of flavonoids on HEL-induced blood flow decrease. The compounds **1**–**3** (10 mg/kg), **4** (10 and 20 mg/kg), and **5**–**13** (20 mg/kg) were administered on days 0, 3, and 6 after sensitization.

In this study, we employed a previously developed *in vivo* assay system to determine whether the leaves and flowers of *S. vulgaris* can prevent blood stasis syndrome.^(10)^ The present data confirm their preventive activities on blood stasis syndrome. The administration of LSV and FSV significantly inhibited HEL-induced blood flow decrease. Among the constituents of LSV, quercetin 3-glucoside (**1**), quercetin 3-rutinoside (**2**), and acteoside (**5**) may play important roles in the prevention of *oketsu*. Flavonoids **1** and **2** have been isolated from many plants, and their various biological effects such as antioxidant, anti-inflammatory, and antiplatelet aggregation activities have been reported.^(14–17)^ However, this is the first study to report the ameliorating effects of LSV and FSV on blood stasis syndrome using HEL-sensitized model mice. We previously reported that the mechanisms of HEL-induced blood flow decrease are complicated and involve various factors that may be related to stagnant blood and inflammation, such as NO, PGI_2_, TXA_2_, ET-1, COX and NOS. ^(11,12)^ There have been many reports on the inhibitory effects of compounds **1** and **2** on the above factors *in vitro* or in another models.^(14,16,19)^ Inhibition of blood flow decrease by compounds **1** and **2** may occur in a similar manner. In contrast, flavonoids **3** and **4**, which have been reported to exert inhibitory effects on the above-mentioned factors,^(14,16,18)^ did not inhibit blood flow decrease. *In vivo* and *in vitro* activities of polyphenols from *S. vulgaris* may not necessarily be similar. Further studies should be performed to investigate the ameliorating effects on blood stasis syndrome *in vivo*. Previously, we reported that quercetin (**6**), kaempferol (**11**), and apigenin 7-glucoside (**13**) did not inhibit HEL-induced blood flow decrease, while quercetin-3-*O*-β-d-xylopyranosyl-(1→2)-*O*-β-d-galactopyranoside (**7**), mutabiloside (**8**), luteolin (**9**), and luteolin 7-glucoside (**10**) significantly did.^(19,20)^ Therefore, quercetin and luteolin glucosides, which feature two hydroxyl groups in the B ring, presented a higher inhibitory effect than kaempferol and apigenin glucosides, which feature only one hydroxyl group in the B ring (Fig. 5). Currently, there are several reports on the absorption mechanism of flavonoids, which exhibits various useful biological activities.^(21–24)^ It is suggested that the absorption mechanism of flavonoids in the gastrointestinal tract varies according to the type and bond position of the sugar; however, in the literature, consensus has not been reached regarding the association of flavonoids with intestinal bacteria and intestinal mucosa enzymes and regarding the existence of a transporter. The existent knowledge on the active expression of flavonoid glucosides should be expanded.

In the present study, we used mice to demonstrate that the flowers and leaves of *S. vulgaris* exerted inhibitory effects on blood stasis syndrome for the first time. To our knowledge, this is a new function of *S. vulgaris*, because the plant had not yet been utilized as a treatment option for *oketsu*. Our results suggest that polyphenols from this plant could be useful for the development of new agents to preventing *oketsu* and could improving the quality of life of individuals with disorders and diseases such as gynecopathy, cold sensitivity, poor circulation, allergy, and lifestyle-related diseases. The mechanism of action and identification of other active compounds are presently under investigation.

## Abbreviations

AcOEt: ethyl acetate
COX: cyclooxygenase
ET: endothelin
HEL: hen egg white lysozyme
iNOS: inducible nitric oxide synthase
*n*-BuOH: *n*-butanol
TX: thromboxane
